# Supplementary material for: Synthetic Nucleic Acid Antigens in Localized Scleroderma
Source: Int J Mol Sci. 2023 Dec 15;24(24):17507. doi: 10.3390/ijms242417507 (PMC10744100; doi:10.3390/ijms242417507)
Supplement: Supplementary file 1 [file ijms-24-17507-s001.zip › ijms-2684861-supplementary.pdf]

# Supporting Information

## Contents

1. Table S1. Genes with reported altered expression in LS. .... 2

2. Patients and controls .... 4

Table S2. Demographic and clinical features of the patients and controls used in human serology study. .... 4

Table S3. Additional clinical features of the LS patients used in human serology study..... 4

3. In house ELISA of human samples .... 5

Figure S1. Results of ELISA with CTD used as antigen, LS (70), SLE (11) and healthy (11), presented as box plot with outliers.. .... 5

4. Statistical analyses – ELISA of human samples .... 6

Figure S2: $Z_1$ matrix describing the repeated measured structure induced by the LS patients. .... 10

5. Study in mice .... 12

Figure S3. Weight chart for animals at different observation points. A) Bleomycin induced C57BL/6J; B) non induced (healthy) C57BL/6J; C) Tamoxifen induced NZB/NZD mice. . 12

6. In house ELISA of mice samples .... 13

1. Table S1. Genes with reported altered expression in LS.*

| Gene | Description | Reference |
|---|---|---|
| *COL8A1* | Expression components of collagen subtypes with direct effect on fibrotic processes. | *Tognetti, L. et al. (2022)* |
| *COL12A1* | | |
| *CTHRC1* | Negative regulator of collagen deposition, being overexpressed in anti-fibrotic processes. | |
| *FBN1* | A duplication within the Fbn1 gene is associated to the mutation used for the thickened skin mice that model LS. | *Arnett, F. C. et al. (1999)* *Siracusa, L. D. et al. (1996)* |
| *IGH* | All these genes were found dysregulated in a microarray hybridization showing a gene expression signature for different scleroderma forms, including morphea. | *Milano, A. et al. (2008)* |
| *IGHG4* | | |
| *IGJ* | | |
| *IGKC* | | |
| *F7* | | |
| *IGLC2* | | |
| *CCL4* | | |
| *MT1X* | | |
| *IGLL1* | | |
| *E2F7* | | |
| *PLAC8* | | |
| *MCM8* | | |
| *let-7a* | Negative regulator of collagen I expression. In LSc patients, let-7a expression is reduced by TGF-β signaling. | *Makino, K. et al. (2013)* |
| *miR483-5p* | Upregulated in autoimmune diseases with fibrotic features, including fibrosis development in LS. | *Chouri, E. et al. (2018)* |
| *miR7* | Its downregulation is linked to excessive collagen expression in LS | *Etoh, M. et al. Res. (2013)* |
| *miRNA-155* | Upregulated in the affected skin of LS patients and proven necessary to induce skin fibrosis in bleomycin mice model. Also, topical treatment with miRNA-155 resulted into an effective treatment for dermal fibrosis in mice models. | *Yan, Q. et al. (2016)* |
| *miRNA-196a* | Negative regulator of collagen I and suspectedly driving pathogenesis. | *Makino, T. et al. (2014)* |
| *BAX* | Anti-apoptotic Bcl-2 family and its associated pro-apoptotic protein BAX levels are increased during the myofibroblast differentiation that occurs during fibrosis. TGF-β1 induced fibrotic myofibroblasts have reported increased levels of both Bcl-2 and BAX mRNAs, and fibrotic bleomycin models of mice have proven higher Bcl-2 concentrations than controls. | *Park, YS. et al. (2019)* *Lagares, D. et al. (2017)* |
| *BCL2* | | |
| *BLK* | Risk locus in SSc and SLE. | *Gourh, P. et al. (2010)* |
| *CCL2* | Marker of inflammatory response. Used with human and murine LS models to follow treatment effects. | *Matthew, B. et al. (2012)* *Badea, I. et al. (2012)* |

| | | |
|---|---|---|
| *CD19* | Overexpressed in B cells of LS affected tissue and directly related to fibrosis in tight skin mice model. | *McNallan, K. T. et al. (2007)* *Saito, E. et al. (2002)* |
| *CD44* | Related to inflammatory response, and highly expressed in LS patients and mice. | *Scuderi, N. et al. (2013)* *Matthew, B. et al. (2012)* |
| *CD52* *LY86* *IRF1* *FGL2* | All these genes were found dysregulated showing a gene expression signature for different scleroderma forms, including LS, in both human and murine models. | *Greenblatt M. B. et al. (2012)* |
| *CRP* | Common marker for inflammatory processes. Overexpressed among a cohort of juvenile morphea, especially for those with the deep subtype. | *Zulian, F. et al. (2006)* |
| *FAS* | Fas pathway induces endothelial cell apoptosis in SSc . Even though further studies need to be reported in LS, bleomycin sclerotic mice model showed increased expression of Fas. Induction of scleroderma was substantially reduced in Fas-deficient mice, showing its key role in the disease. | *Stummvoll, G. H. et al. (2000)* *Yamamoto, T. et al. (2007)* |
| *FCGR3* | Fc Gamma receptors have a role in autoimmunity, specifically recognition and processing of IgGs and immune complexes. FCGR3 is dysregulated for different scleroderma forms, including LS. Moreover, autoantibodies for this receptor have been found in morphea patients. | *Milano, A. et al. (2008)* *Davis, K. et al. (1995)* |
| *Icam1* | ICAM-1 plays a role on the leukocyte adhesion to the endothelium and its transport to the tissues where the inflammatory process takes place. LS patients express ICAM-1 at the affected sites with infiltrating mononuclear cells, as well as counting with elevated blood levels of it. | *Majewski, S. et al. (1991)* *Ihn, N. et al. (1994)* |
| *VCAM-1* | Related to leucocyte transport and is upregulated both in human LS and sclerotic mouse. | *J.S. Mertens, et al. (2019)* *L. Zhou, et al. (2007)* |
| *IFN-γ* *IL4* *IL6* | As an autoimmune disease, LS is considered to be propagated by an imbalance of cytokines IFN-γ, IL-4 and IL-6 levels were found dysregulated, being these two last ones inversely correlated with LoSDI. | *Torok, K. S. et al. (2015)* *Ihn, H. et al. (1995)* |
| *ISG15* | Produced by type I IFN signaling and as a response to antiviral infection, and found overexpressed in LS patients. | *Mirizio, E. et al. (2021)* |
| *KRASs* | Pathway related to cell proliferation, migration, differentiation, apoptosis and tissue T cell infiltration. Found overexpressed in morphea patients, especially in an active disease subgroup. | |
| *MZB1* | Proinflammatory agent implicated with the outcome of chronic inflammation and correlated positively with tissue IgG levels. Overexpressed in LS patients. | *HB. Schiller, et al. Am. J. Resp. Crit. Care (2017)* |

* Genes reported in humans; genes with confirmed effect in LS mice model are shown in green.[1]

## 2. Patients and controls

Table S2. Demographic and clinical features of the patients and controls used in human serology study.*

| Parameter | Healthy | LS | SLE** |
|---|---|---|---|
| Number of individuals | 11 | 70 | 11 |
| Female, *n* (%) | 8 (73) | 56 (80) | 8 (73) |
| Age at sample, median (range) | 19 (18-23) | 14.6 (2.5-25.1) | 17.0 (10.1-20.2) |
| Race, white *n* (%) | 11 (100) | 70 (100) | 10 (91) |
| Race, african-american *n* (%) | 0 (0) | 0 (0) | 1 (9) |
| Ethnicity, hispanic, *n* (%) | 2 (18) | 1 (9) | 3 (27) |
| Disease activity, mean value (range) | n/a | 0.3 (0-1) | 5 (0-9) |
| ANA positive *n* (%), IFA | n/a | n/d | 7 (64) |
| Anti-dsDNA positive *n* (%), IFA | n/a | n/d | 6 (55) |
| Anti-ssDNA positive *n* (%), ELISA | n/a | 7 (10) | 8 (73) |
| Anti-histone positive, *n* (%), ELISA | n/a | 16 (23) | 5 (45) |

* n/a not applicable, n/d no data. ** SLEDAI is used as disease activity index.

Table S3. Additional clinical features of the LS patients used in human serology study.*

| Parameter | LS |
|---|---|
| mLOSSI median (range) | 1 (0-39) |
| LOSAI median (range) | 0 (0-20) |
| mLOSDI median (range) | 7 (0-110) |
| PGAACT median (range) | 0 (0-96) |
| PGADAMG median (range) | 32 (0-77) |
| Aldolase median (range) | 5 (0-13) |
| White blood cells median (range) | 6.8 (1-18) |
| Lymphocytes median (range) | 31 (0-81) |
| Monocytes median (range) | 7 (0-15) |
| Eosinophils median (range) | 2 (0-15) |
| ESR median (range) | 7 (0-42) |
| CRP median (range) | 0.04 (0-6.6) |

* Abbreviations: mLOSSI = modified Localized *Scleroderma* Skin Severity Index, LOSAI = Localized *Scleroderma* Activity Index, mLOSDI = modified Localized *Scleroderma* Damage Index, PGAACT = physicians' global assessment of disease activity, PGADAMG = physicians' global assessment of disease damage, ESR = erythrocyte sedimentation rate, CRP = C-reactive protein.

## 3. In house ELISA of human samples

Antigens have been synthesized in house and characterized as previously described.[1] General protocol for in house ELISA is given below.

**Antigens.** Calf thymus DNA (CTD) from Sigma-Aldrich (cat no 2618, Copenhagen, Denmark) was used as specificity control. Single stranded antigens were used without prior annealing.

Single/Double stranded DNA/LNA/RNA antigen or CTD for single ELISA plate were applied one per well. For duplexes, single stranded complementary strands were annealed in 1:1 molar ratio in 10X PBS buffer for 10 minutes at 92 °C, followed by colling to room temperature over 30 min. The volume was then restored in 1X PBS to get the final concentration of antigen 3.5 µg/mL.

**Secondary antibodies (conjugated antisera):** Species specific (human, mice), IgG (Fc specific), IgM (µ-chain specific), IgA and IgE (ε-chain specific) developed in goat or rabbit were used as secondary antibodies (Sigma-Aldrich, Copenhagen, Denmark). HRP antibody solution was prepared by diluting commercially available anti-serum conjugate in previously prepared diluent (2 g BSA, 50 µL Tween-20, 1L 1X PBS), in v/v ratio 1:20,000.

### Enzyme Linked Immunosorbent Assay (ELISA)

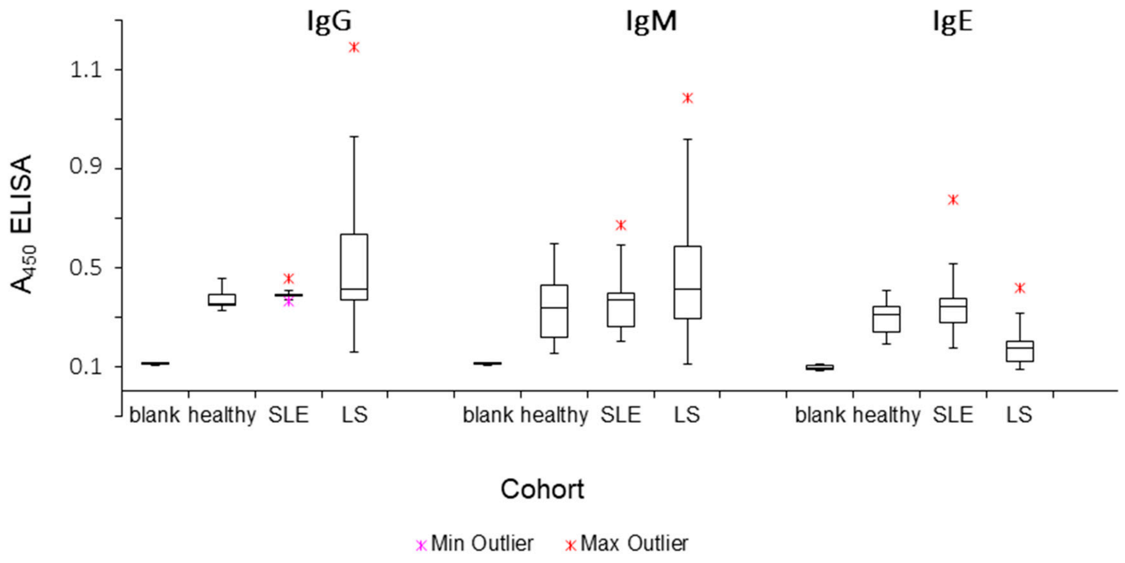


**Figure S1. Results of ELISA with CTD used as antigen, LS (70), SLE (11) and healthy (11), presented as box plot with outliers (marked as stars).** Each measurement was done in duplicate; mean values for intra-study duplicate are presented.

[1] Khatri, Astakhova et al. Methods Mol Biol Springer, 2020.

## 4. Statistical analyses – ELISA of human samples

Multiple (pairwise) comparisons using Tukey's Honest Significant Difference (HSD) test:

| Difference | Estimate | SE | LCB | UCB | p-value |
|---|---|---|---|---|---|
| LS - SLE | 0.1506 | 0.0410 | 0.0436 | 0.258 | 0.0021 |
| LS - Healthy | 0.1785 | 0.0410 | 0.0715 | 0.286 | 0.0002 |
| LS - Blank | 0.3472 | 0.0395 | 0.2441 | 0.450 | < 0.0001 |
| SLE - Healthy | 0.0279 | 0.0539 | -0.1128 | 0.169 | 0.9545 |
| SLE - Blank | 0.1965 | 0.0527 | 0.0588 | 0.334 | 0.0018 |
| Healthy - Blank | 0.1686 | 0.0527 | 0.0309 | 0.306 | 0.0099 |

Table S4: All pairwise comparisons of group means, (ds)D4, IgG ELISA.

| | Estimate |
|---|---|
| LS | $0.3870_{c}$ |
| SLE | $0.2364_{b}$ |
| Healthy | $0.2085_{b}$ |
| Blank | $0.0398_{a}$ |

Table S5: Summarizing table of means using compact letter displays, (ds)D4, IgG ELISA.

| Difference | Estimate | SE | LCB | UCB | p-value |
|---|---|---|---|---|---|
| LS - SLE | 0.2937 | 0.0454 | 0.17519 | 0.4122 | < 0.0001 |
| LS - Healthy | 0.2373 | 0.0454 | 0.11873 | 0.3558 | < 0.0001 |
| LS - Blank | 0.3868 | 0.0437 | 0.27260 | 0.5009 | < 0.0001 |
| SLE - Healthy | -0.0565 | 0.0596 | -0.21228 | 0.0994 | 0.7798 |
| SLE - Blank | 0.0931 | 0.0584 | -0.05948 | 0.2456 | 0.3867 |
| Healthy - Blank | 0.1495 | 0.0584 | -0.00302 | 0.3021 | 0.0569 |

Table S6: All pairwise comparisons of group means, (ds)D5, IgG ELISA.

| | Estimate |
|---|---|
| LS | $0.4324_{b}$ |
| SLE | $0.1387_{a}$ |
| Healthy | $0.1952_{a}$ |
| Blank | $0.0457_{a}$ |

Table S7: Summarizing table of means using compact letter displays, (ds)D5, IgG ELISA.

| Difference | Estimate | SE | LCB | UCB | p-value |
|---|---|---|---|---|---|
| LS - SLE | 0.2138 | 0.0451 | 0.0959 | 0.332 | < 0.0001 |
| LS - Healthy | 0.3198 | 0.0451 | 0.2019 | 0.438 | < 0.0001 |
| LS - Blank | 0.4184 | 0.0435 | 0.3048 | 0.532 | < 0.0001 |
| SLE - Healthy | 0.1060 | 0.0593 | -0.0490 | 0.261 | 0.2857 |
| SLE - Blank | 0.2046 | 0.0581 | 0.0529 | 0.356 | 0.0036 |
| Healthy - Blank | 0.0986 | 0.0581 | -0.0531 | 0.250 | 0.3303 |

Table S8: All pairwise comparisons of group means, (ds)HUV, IgG ELISA.

| | Estimate |
|---|---|
| LS | $0.4529_{c}$ |
| SLE | $0.2391_{b}$ |
| Healthy | $0.1331_{ab}$ |
| Blank | $0.0345_{a}$ |

Table S9: Summarizing table of means using compact letter displays, (ds)HUV, IgG ELISA.

| Difference | Estimate | SE | LCB | UCB | p-value |
|---|---|---|---|---|---|
| LS - SLE | 0.2846 | 0.0440 | 0.1698 | 0.399 | < 0.0001 |
| LS - Healthy | 0.3116 | 0.0440 | 0.1967 | 0.426 | < 0.0001 |
| LS - Blank | 0.3952 | 0.0423 | 0.2846 | 0.506 | < 0.0001 |
| SLE - Healthy | 0.0269 | 0.0578 | -0.1241 | 0.178 | 0.9664 |
| SLE - Blank | 0.1105 | 0.0566 | -0.0373 | 0.258 | 0.2125 |
| Healthy - Blank | 0.0836 | 0.0566 | -0.0642 | 0.231 | 0.4543 |

Table S10: All pairwise comparisons of group means, (ds)L3D, IgG ELISA.

| | Estimate |
|---|---|
| LS | $0.432_{b}$ |
| SLE | $0.148_{a}$ |
| Healthy | $0.121_{a}$ |
| Blank | $0.037_{a}$ |

Table S11: Summarizing table of means using compact letter displays, (ds)L3D, IgG ELISA.

| Difference | Estimate | SE | LCB | UCB | p-value |
|---|---|---|---|---|---|
| LS - SLE | 0.1906 | 0.0483 | 0.0643 | 0.317 | 0.0008 |
| LS - Healthy | 0.2720 | 0.0483 | 0.1457 | 0.398 | < 0.0001 |
| LS - Blank | 0.4067 | 0.0466 | 0.2851 | 0.528 | < 0.0001 |
| SLE - Healthy | 0.0815 | 0.0635 | -0.0846 | 0.247 | 0.5765 |
| SLE - Blank | 0.2162 | 0.0622 | 0.0536 | 0.379 | 0.0042 |
| Healthy - Blank | 0.1347 | 0.0622 | -0.0278 | 0.297 | 0.1400 |

Table S12: All pairwise comparisons of group means, ssD4, IgG ELISA.

| | Estimate |
|---|---|
| LS | $0.4605_{c}$ |
| SLE | $0.2699_{b}$ |
| Healthy | $0.1885_{ab}$ |
| Blank | $0.0537_{a}$ |

Table S13: Summarizing table of means using compact letter displays, ssD4, IgG ELISA.

| Difference | Estimate | SE | LCB | UCB | p-value |
|---|---|---|---|---|---|
| LS - SLE | 0.2316 | 0.0493 | 0.1028 | 0.360 | < 0.0001 |
| LS - Healthy | 0.2958 | 0.0493 | 0.1670 | 0.425 | < 0.0001 |
| LS - Blank | 0.4306 | 0.0475 | 0.3065 | 0.555 | < 0.0001 |
| SLE - Healthy | 0.0642 | 0.0648 | -0.1051 | 0.233 | 0.7552 |
| SLE - Blank | 0.1989 | 0.0634 | 0.0332 | 0.365 | 0.0119 |
| Healthy - Blank | 0.1348 | 0.0634 | -0.0310 | 0.301 | 0.1524 |

Table S14: All pairwise comparisons of group means, ssLS_ag1, IgG ELISA.

| | Estimate |
|---|---|
| LS | $0.4670_{c}$ |
| SLE | $0.2354_{b}$ |
| Healthy | $0.1712_{ab}$ |
| Blank | $0.0364_{a}$ |

Table S15: Summarizing table of means using compact letter displays, ssLS_ag1, IgG ELISA.

| Difference | Estimate | SE | LCB | UCB | p-value |
|---|---|---|---|---|---|
| LS - SLE | 0.115 | 0.0994 | -0.14522 | 0.374 | 0.6582 |
| LS - Healthy | -0.391 | 0.0994 | -0.65113 | -0.132 | 0.0009 |
| LS - Blank | 0.449 | 0.0994 | 0.18969 | 0.709 | 0.0001 |
| SLE - Healthy | -0.506 | 0.1307 | -0.84741 | -0.164 | 0.0011 |
| SLE - Blank | 0.335 | 0.1307 | -0.00659 | 0.676 | 0.0567 |
| Healthy - Blank | 0.841 | 0.1307 | 0.49932 | 1.182 | < 0.0001 |

Table S16: All pairwise comparisons of group means, (ds)D4, IgM ELISA.

| | Estimate |
|---|---|
| LS | $0.5221_{c}$ |
| SLE | $0.4075_{ac}$ |
| Healthy | $0.9135_{b}$ |
| Blank | $0.0726_{a}$ |

Table S17: Summarizing table of means using compact letter displays, (ds)D4, IgM ELISA.

| Difference | Estimate | SE | LCB | UCB | p-value |
|---|---|---|---|---|---|
| LS - SLE | 0.2446 | 0.0366 | 0.1490 | 0.340 | < 0.0001 |
| LS - Healthy | 0.2653 | 0.0366 | 0.1697 | 0.361 | < 0.0001 |
| LS - Blank | 0.3219 | 0.0366 | 0.2263 | 0.417 | < 0.0001 |
| SLE - Healthy | 0.0207 | 0.0481 | -0.1049 | 0.146 | 0.9730 |
| SLE - Blank | 0.0773 | 0.0481 | -0.0484 | 0.203 | 0.3796 |
| Healthy - Blank | 0.0565 | 0.0481 | -0.0691 | 0.182 | 0.6435 |

Table S18: All pairwise comparisons of group means, (ds)D5, IgM ELISA.

| | Estimate |
|---|---|
| LS | $0.3988_{b}$ |
| SLE | $0.1542_{a}$ |
| Healthy | $0.1335_{a}$ |
| Blank | $0.0769_{a}$ |

Table S19: Summarizing table of means using compact letter displays, (ds)D5, IgM ELISA.

| Difference | Estimate | SE | LCB | UCB | p-value |
|---|---|---|---|---|---|
| LS - SLE | 0.1871 | 0.0387 | 0.08595 | 0.288 | < 0.0001 |
| LS - Healthy | 0.2786 | 0.0387 | 0.17741 | 0.380 | < 0.0001 |
| LS - Blank | 0.3263 | 0.0387 | 0.22514 | 0.427 | < 0.0001 |
| SLE - Healthy | 0.0915 | 0.0509 | -0.04154 | 0.224 | 0.2809 |
| SLE - Blank | 0.1392 | 0.0509 | 0.00619 | 0.272 | 0.0366 |
| Healthy - Blank | 0.0477 | 0.0509 | -0.08527 | 0.181 | 0.7846 |

Table S20: All pairwise comparisons of group means, (ds)HUV, IgM ELISA.

| | Estimate |
|---|---|
| LS | $0.4048_{c}$ |
| SLE | $0.2177_{b}$ |
| Healthy | $0.1263_{ab}$ |
| Blank | $0.0785_{a}$ |

Table S21: Summarizing table of means using compact letter displays, (ds)HUV, IgM ELISA.

| Difference | Estimate | SE | LCB | UCB | p-value |
|---|---|---|---|---|---|
| LS - SLE | 0.0503 | 0.0282 | -0.0234 | 0.124 | 0.2874 |
| LS - Healthy | 0.1207 | 0.0282 | 0.0470 | 0.194 | 0.0003 |
| LS - Blank | 0.1582 | 0.0282 | 0.0845 | 0.232 | < 0.0001 |
| SLE - Healthy | 0.0704 | 0.0371 | -0.0265 | 0.167 | 0.2358 |
| SLE - Blank | 0.1079 | 0.0371 | 0.0110 | 0.205 | 0.0228 |
| Healthy - Blank | 0.0375 | 0.0371 | -0.0594 | 0.134 | 0.7425 |

Table S22: All pairwise comparisons of group means, dsLS_ag2, IgE ELISA.

| | Estimate |
|---|---|
| LS | $0.2495_{c}$ |
| SLE | $0.1992_{bc}$ |
| Healthy | $0.1288_{ab}$ |
| Blank | $0.0913_{a}$ |

Table S23: Summarizing table of means using compact letter displays, dsLS_ag2, IgE ELISA.

| Difference | Estimate | SE | LCB | UCB | p-value |
|---|---|---|---|---|---|
| LS - SLE | 0.0370 | 0.0268 | -0.0330 | 0.107 | 0.5148 |
| LS - Healthy | 0.1092 | 0.0268 | 0.0392 | 0.179 | 0.0005 |
| LS - Blank | 0.1494 | 0.0268 | 0.0764 | 0.216 | < 0.0001 |
| SLE - Healthy | 0.0723 | 0.0352 | -0.0197 | 0.164 | 0.1761 |
| SLE - Blank | 0.1095 | 0.0352 | 0.0174 | 0.201 | 0.0129 |
| Healthy - Blank | 0.0372 | 0.0352 | -0.0548 | 0.129 | 0.7170 |

Table S24: All pairwise comparisons of group means, EBF3_r, IgE ELISA.

| | Estimate |
|---|---|
| LS | $0.247_{c}$ |
| SLE | $0.210_{bc}$ |
| Healthy | $0.138_{ab}$ |
| Blank | $0.101_{a}$ |

Table S25: Summarizing table of means using compact letter displays, EBF3_r, IgE ELISA.

| Difference | Estimate | SE | LCB | UCB | p-value |
|---|---|---|---|---|---|
| LS - SLE | 0.0294 | 0.0264 | -0.03973 | 0.0985 | 0.6838 |
| LS - Healthy | 0.1115 | 0.0264 | 0.04236 | 0.1806 | 0.0003 |
| LS - Blank | 0.1378 | 0.0264 | 0.06873 | 0.2069 | < 0.0001 |
| SLE - Healthy | 0.0821 | 0.0348 | -0.00875 | 0.1729 | 0.0914 |
| SLE - Blank | 0.1085 | 0.0348 | 0.01762 | 0.1993 | 0.0125 |
| Healthy - Blank | 0.0264 | 0.0348 | -0.06448 | 0.1172 | 0.8729 |

Table S26: All pairwise comparisons of group means, ssD4, IgE ELISA.

| | Estimate |
|---|---|
| LS | $0.2345_{c}$ |
| SLE | $0.2051_{bc}$ |
| Healthy | $0.1230_{ab}$ |
| Blank | $0.0966_{a}$ |

Table S27: Summarizing table of means using compact letter displays, ssD4, IgE ELISA.

A covariance generalized linear model (cGLM) fitted to IgG ELISA data:

Call: DiseaseScore ~ (ds)D4 + (ds)HUV + ssD4

| | **Estimate** | **Standard error** | **Z-value** | **p-value** |
|---|---|---|---|---|
| (Intercept) | -10.0027243 | 1.255004 | -7.9702725 | < 0.001 |
| (ds)D4 | 16.7747376 | 2.486166 | 6.7472323 | < 0.001 |
| (ds)HUV | 0.1510628 | 1.449917 | 0.1041872 | 0.9170208 |
| ssD4 | 4.4121399 | 1.487825 | 2.9654969 | 0.0030219 |

Table S28: Estimated regression parameters.

| | **Estimate** | **Standard error** | **Z-value** | **p-value** |
|---|---|---|---|---|
| $\tau_0$ | 0.4797388 | 0.17667841 | 2.715322 | 0.0066211 |
| $\tau_1$ | 0.1615470 | 0.07091444 | 2.278056 | 0.0227233 |

Table S29: Estimated dispersion parameters.

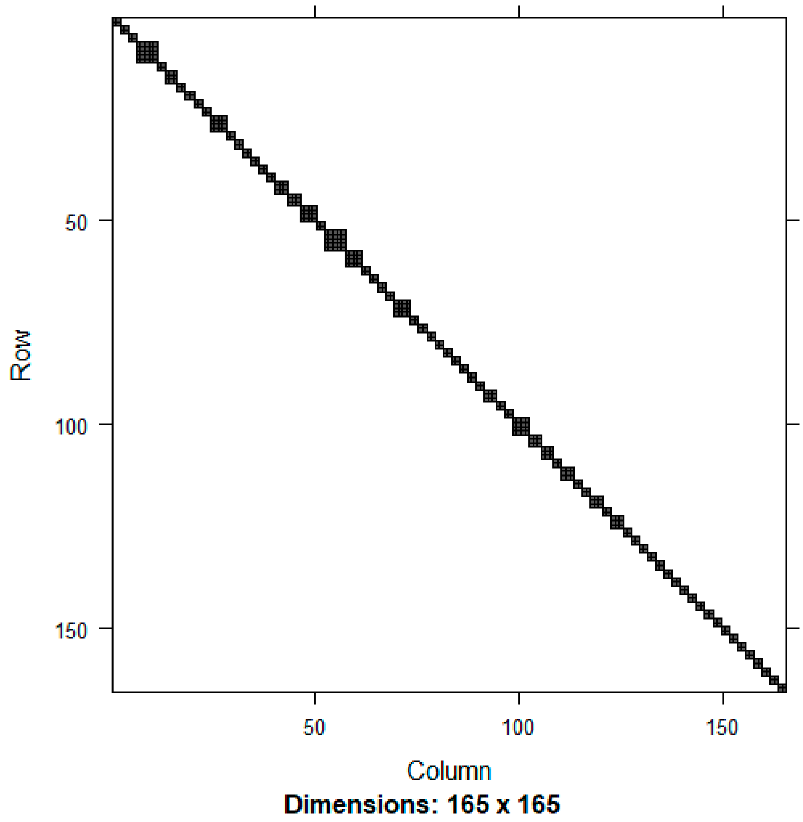


Figure S2: $Z_1$ matrix describing the repeated measured structure induced by the LS patients.

The covariate (ds)HUV is eliminated:

Call: DiseaseScore ~ (ds)D4 + ssD4

| | **Estimate** | **Standard error** | **Z-value** | **p-value** |
|---|---|---|---|---|
| (Intercept) | -9.990769 | 1.248250 | -8.003819 | < 0.001 |
| (ds)D4 | 16.799721 | 2.457225 | 6.836867 | < 0.001 |
| ssD4 | 4.508600 | 1.327793 | 3.395559 | < 0.001 |

Table S30: Estimated regression parameters.

| | **Estimate** | **Standard error** | **Z-value** | **p-value** |
|---|---|---|---|---|
| $\tau_0$ | 0.4835992 | 0.18197907 | 2.657444 | 0.0078736 |
| $\tau_1$ | 0.1636836 | 0.07178392 | 2.280227 | 0.0225942 |

Table S31: Estimated dispersion parameters.

## 5. Study in mice

A)

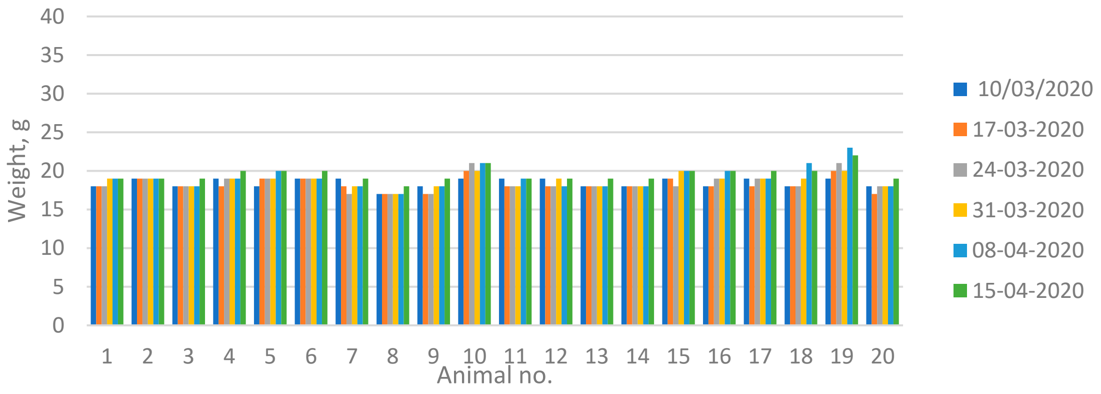


B)

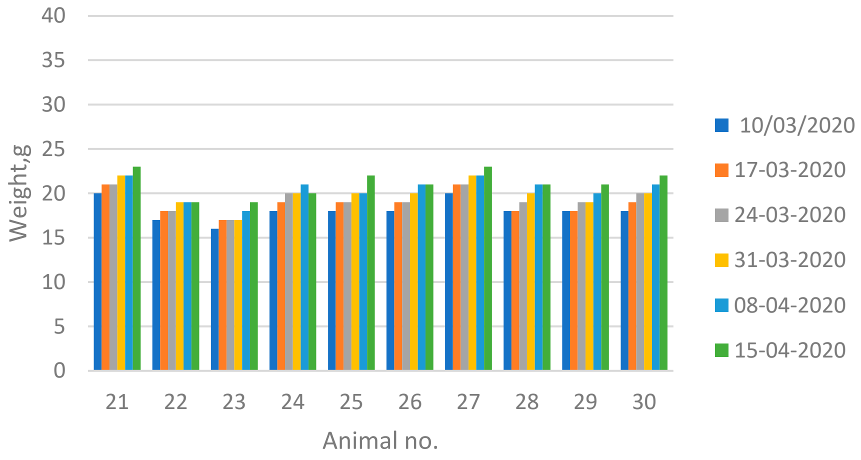


C)

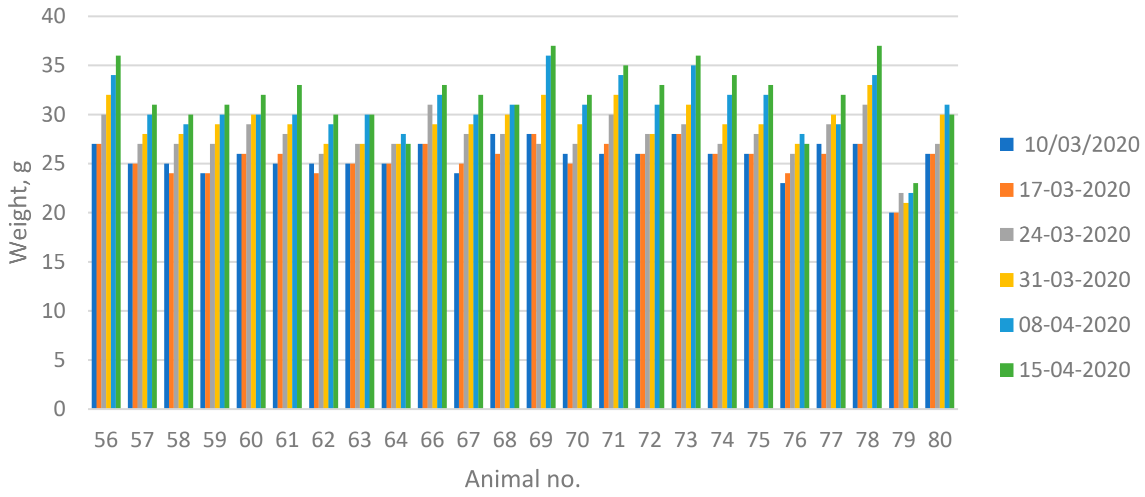


**Figure S3. Weight chart for animals at different observation points.** A) Bleomycin induced C57BL/6J; B) non induced (healthy) C57BL/6J; C) Tamoxifen induced NZB/NZD mice.

## 6. In house ELISA of mice samples

Table S32. Serological analyses of mice sera samples - IgG.*

| Animal group and Antigen | | ELISA antigen/Data point** | | | | | | | | | |
|---|---|---|---|---|---|---|---|---|---|---|---|
| | | dsD4 A** | dsD4 O | dsD5 A | dsD5 O | dsHUV A | dsHUV O | Ag1 A | Ag1 O | PLox A | PLox O |
| Bleomycin LS | dsD4 L | 0.035 | 0.074 | 0.070 | 0.079 | 0.033 | 0.077 | 0.056 | 0.069 | 0.047 | 0.103 |
| | dsD4 H | 0.039 | 0.089 | 0.091 | 0.084 | 0.045 | 0.100 | 0.068 | 0.083 | 0.068 | 0.175 |
| | dsD5 L | 0.087 | 0.071 | 0.125 | 0.075 | 0.076 | 0.067 | 0.068 | 0.074 | 0.066 | 0.175 |
| | dsD5 H | 0.076 | 0.072 | 0.102 | 0.066 | 0.046 | 0.067 | 0.057 | 0.070 | 0.053 | 0.163 |
| NZB/NZW tamoxifen SLE | dsD4 L | 0.079 | 0.098 | 0.137 | 0.084 | 0.069 | 0.089 | 0.047 | 0.025 | 0.115 | 0.154 |
| | dsD4 H | 0.093 | 0.124 | 0.154 | 0.128 | 0.078 | 0.129 | 0.058 | 0.030 | 0.132 | 0.161 |
| | dsD5 L | 0.114 | 0.088 | 0.131 | 0.085 | 0.092 | 0.065 | 0.047 | 0.000 | 0.111 | 0.193 |
| | dsD5 H | 0.107 | 0.100 | 0.146 | 0.094 | 0.104 | 0.081 | 0.050 | 0.048 | 0.118 | 0.232 |
| Healthy | dsD4 L | 0.058 | 0.052 | 0.098 | 0.059 | 0.057 | 0.058 | 0.070 | 0.083 | 0.055 | 0.152 |
| | dsD4 H | 0.056 | 0.061 | 0.095 | 0.051 | 0.059 | 0.063 | 0.060 | 0.098 | 0.054 | 0.138 |
| | dsD5 L | 0.061 | 0.050 | 0.075 | 0.059 | 0.043 | 0.041 | 0.066 | 0.103 | 0.053 | 0.178 |
| | dsD5 H | 0.058 | 0.063 | 0.122 | 0.058 | 0.076 | 0.056 | 0.082 | 0.112 | 0.058 | 0.144 |

* A450 values of ELISA are given as mean values for 5 mice per each group. L = low dose; H = high dose. Mean A450 value given for each group (average for 5 animals). **Data point: A = end of administration; O = end of observation.

Table S33. Serological analyses of mice sera samples – IgM.*

| Animal group and Antigen | | ELISA antigen/Data point** | | | | | | | | | |
|---|---|---|---|---|---|---|---|---|---|---|---|
| | | dsD4 A** | dsD4 O | dsD5 A | dsD5 O | dsHUV A | dsHUV O | Ag1 A | Ag1 O | PLox A | PLox O |
| Bleomycin LS | dsD4 L | 0.097 | 0.045 | 0.139 | 0.042 | 0.098 | 0.047 | 0.074 | 0.045 | 0.068 | 0.068 |
| | dsD4 H | 0.161 | 0.056 | 0.177 | 0.057 | 0.115 | 0.055 | 0.100 | 0.062 | 0.091 | 0.137 |
| | dsD5 L | 0.154 | 0.049 | 0.171 | 0.049 | 0.125 | 0.053 | 0.101 | 0.040 | 0.118 | 0.113 |
| | dsD5 H | 0.146 | 0.046 | 0.193 | 0.043 | 0.135 | 0.047 | 0.090 | 0.040 | 0.086 | 0.122 |
| NZB/NZW tamoxifen SLE | dsD4 L | 0.123 | 0.124 | 0.173 | 0.123 | 0.110 | 0.125 | 0.091 | 0.118 | 0.128 | 0.114 |
| | dsD4 H | 0.115 | 0.137 | 0.174 | 0.138 | 0.127 | 0.134 | 0.084 | 0.097 | 0.138 | 0.198 |
| | dsD5 L | 0.201 | 0.084 | 0.263 | 0.084 | 0.274 | 0.094 | 0.082 | 0.086 | 0.195 | 0.130 |
| | dsD5 H | 0.226 | 0.127 | 0.290 | 0.134 | 0.301 | 0.131 | 0.079 | 0.095 | 0.182 | 0.171 |
| Healthy | dsD4 L | 0.080 | 0.051 | 0.188 | 0.027 | 0.085 | 0.041 | 0.118 | 0.037 | 0.072 | 0.097 |
| | dsD4 H | 0.092 | 0.043 | 0.144 | 0.031 | 0.090 | 0.047 | 0.074 | 0.037 | 0.065 | 0.097 |
| | dsD5 L | 0.101 | 0.038 | 0.139 | 0.037 | 0.096 | 0.036 | 0.080 | 0.042 | 0.071 | 0.098 |
| | dsD5 H | 0.099 | 0.040 | 0.152 | 0.034 | 0.090 | 0.043 | 0.103 | 0.042 | 0.086 | 0.117 |

* A450 values of ELISA are given as mean values for 5 mice per each group. L = low dose; H = high dose. Mean A450 value given for each group (average for 5 animals). **Data point: A = end of administration; O = end of observation.
